# Influences of phosphorus and foliar iron fertilization rate on the quality parameters of whole wheat grain

**DOI:** 10.1002/fsn3.804

**Published:** 2019-02-03

**Authors:** Feizollah Shahbazi, Ameneh Nematollahi

**Affiliations:** ^1^ Lorestan University Khoram Abad Iran

**Keywords:** iron fertilization, phosphorus, quality, wheat

## Abstract

The objective of this research was to study the effects of phosphorous and foliar iron fertilizations rates on the of protein, fiber, and water contents in wheat grains. Treatments of fertilizers included a combination of three foliar iron rates (0, 1.2, and 2 L/ha, Fusion) and four phosphorus rates (0, 75, 150, and 225 kg/ha, P_2_O_5_) at three replications. The quality parameters of harvested seeds were measured by the NIR technique. Protein content increased but and fiber and water contents decreased significantly with an increase in the iron and phosphorus fertility rates. As the rates of foliar iron increased from 0 to 2 L/ha the mean values of protein content increased significantly from 13.47% to 15.48%. With increasing the rates of phosphorus from 0 to150 kg/ha, the mean values of protein content increased significantly from 13.56% to 15.53%, however, further increase in phosphorus, causing a decreased trend in the amount of protein. Fiber and water contents of wheat decreased significantly from 13.76% to 11.86% and from 7.36% to 7.28%, respectively, as the phosphorous rate increased from 0 to 225 kg/ha. Those changes were from 13.40% to 12.03% and from 7.57% to 7.21%, respectively, for increasing foliar iron from 0, 1.2, and 2 L/ha. Mathematical relationships composed of phosphorus and iron fertilizers were developed for accurately describing the wheat protein, fiber, and water contents under different fertilization rates.

## 1 INTRODUCTION

Wheat (*Triticum aesitivum* L.) species cultivated for over 10,000 years as human food crops and represented the major factor of social, population, and cultural development (Popovic, 2015). Besides the rice, the common or bread wheat is the main human food crop. Just like the other cereals, wheat ensures energy as a concentrated source of carbohydrates (such as starch), protein, fat, mineral, vitamins, and fiber. The protein content of wheat seed widely varied in the amount from 8% to 20%, between the type and classes of wheat (Popovic, 2015).

Wheat seed nutritional quality parameters are strongly dependent on the conditions of the growing, the fertility of soil, fertilizer practice, water availability, genotype, grain handling and storage conditions (Carson & Edwards, 2009; Jayas, Ghosh, Paliwal, & Karunakarna, 2008; Popovic, 2015; Uhlen et al., 1998). Some of the importances of the wheat grains are the grain cover, grain structure, and chemical composition (Shahbazi, Sharafi, Moomevandi, & Daneshvar, 2015). The quality factors are affected by the level of mineral fertilization (Shahbazi, Sharafi, Moomevandi, & Daneshvar, 2014).

Many of today’s cereal seeds such as wheat production environments are managed at a very high application of chemical fertilizers to return plant nutrient to agricultural lands and ensure maximum yield potential. Consequently, it is important to understand the seed properties and quality parameters in response to chemical fertilizers such as phosphorus and iron (Shahbazi et al., 2014).

The 17 elements of balanced fertilization include macronutrients (such as P) and microelements (such as Fe) are required for the wheat crop (Havlin, Beaton, Tisdale, & Nelson, 2005). Phosphorus is important for storage and transfers the energy in plant cells and is essential for the photosynthesis (Taiz & Zeiger, 2010). Phosphorus also stimulates root development and helps plants to be early established in the season (Popovic, 2015). Iron is used to increase concentration and ability of Fe in commonly‐eaten cereal‐based crops such as wheat to improve human nutrition (Shahbazi et al., 2015).

To reach the goal and increase the grain production of wheat with optimal quality for food, measurements and knowledge need to be improved (Popovic, 2015). This will be a great challenge for each member of the chain of food production from farmers to consumers exposed to wheat of variable quality (Popovic, 2015). Improving the knowledge of quality traits in crop program and management practices in fertilizers application techniques will reduce variation in the quality of the wheat grain and secure expected higher quality (Popovic, 2015).

Near‐infrared reflectance (NIR) technology as a rapid, accurate, automated, non‐destructively, and on‐line systems has been used to determine and monitor the chemical composition and physicochemical properties of wheat (Arazuri et al., 2012; Jayas et al., 2008; Sissons, Osborne, & Sissons, 2006).

Many studies have been conducted to determine and predict a number of quality parameters of wheat grain or flour use the NIR measurement such as: moisture, protein, linoleic acid and palmitic acid contents (Delwiche & Hruschka, 2000; Wang, Dowell, & Dempster,^2002^; Delwiche, 2003; Bordes, Branlard, Oury, Charmet, & Balfourier, 2008; : Arazuri et al., 2012), hardness, flour yield, damaged starch, water absorption, dough development time, extensibility, load volume and changes during mixing (Alava, Millar, & Salmon, 2001; Arazuri et al., 2012; Kaddour, Barron, Robert, & Cuq, 2008; Kaddour & Cuq, 2009; Kaddour, Morel, & Cuq, 2008), and durum wheat adulteration (Cocchi et al., 2006).

Little published literature investing the effects of iron and phosphorus fertility on the wheat grain quality parameters is available. Therefore, the objective of this research was to study the impact of phosphorus (0, 75, 150, and 225 kg/ha, P_2_O_5_) and foliar iron (0, 1.2, and 2 L/ha, Fusion) fertilization rates on wheat quality parameters focus protein, fiber, and water content in whole grain wheat seed by using NIR technology.

## 2 MATERIALS AND METHODS

Information about wheat seed cultivar, cropping system, crop management practices, the properties of the studied field soil, and the treatment method combinations of phosphorus and foliar iron rates are described by Shahbazi et al. (2014, 2015). Table 1 shows the physical and chemical properties of the examined field soil.

**Table 1:** Physical and chemical properties of studied soil (0‐15 cm depth)

Soil texture	Soil particles (%)			Total nitrogen (%)	Organic carbon (%)	Available phosphorus (P.P.m)	Available potassium (P.P.m)	Cu (P.P.m)	Fe (P.P.m)	Mn (P.P.m)	Zn (P.P.m)
	Clay	Silt	Sand								
S.C.L	37	44	19	0.09	0.78	8.1	231	0.69	7.8	6.7	0.79

After optimum maturity, samples of grains harvested by hand from each plot, cleaned in an air screen cleaner, sealed in polyethylene bags, and then stored in a humid environment for 6 months to standardize their moisture content (Shahbazi, 2012). The initial moisture content of seed treatments in the binging of storage was about 10.68% (wet basis) determined with ASAE S352.2 (ASAE Standards, 1990).

Wheat quality parameters of whole wheat grain include protein content, fiber content (neutral detergent fiber [NDF]), and water content (stored water for 6 months) were measured by near‐infrared reflectance (NIR) technique for whole grains by using Perten DA 7250 NIR analyzer instrument (Perten Instruments AB, Huddinge, Sweden).

In this study, the effects of phosphorus fertilizer rates (0, 75, 150, and 225 kg/ha) and foliar iron fertilizer rate (0, 1.2, and 2 L/ha) were studied on the wheat quality parameters. The factorial experiment was conducted as a randomized design with three replicates. For each quality parameter test, required wheat grains were selected randomly from each sample and tested by using NIR analyzer instrument. The main treatments and their interactions analyzed using analysis of variance (ANOVA) by using SPSS software; version 19 for graphs and tables Microsoft Excel was used. Level of significance was shown as *p < 0.05 and **p < 0.01 applying Duncan's multiple range tests.

## 3 RESULTS AND DISCUSSION

The results of variance analysis showed the effects of the two independent variables, namely, phosphorus and foliar iron rates on the protein content of wheat grains were significant at 1% probability level (*p* < 0.01) (Table 2). The effect of phosphorus rate on the variation in the protein content of wheat grains was higher (*F* = 84.06) than the iron rate (*F* = 23.03), within the ranges of studied variables (Table 2). Also, the interaction effect of the phosphorus and foliar iron rate on the percent protein of grains was significant at 5% probability level (*p* < 0.05). In addition, the rate of two fertilizers significantly influenced the fiber content of whole wheat grains at 1% probability level (Table 2), phosphorus rate had the most (*F* = 24.32) but, the iron rate had the least (*F* = 22.02) influences on the fiber content. The interaction effect phosphorus and foliar iron fertilizers rate on the fiber content of seeds was not significant. The rates of both phosphorus and foliar iron fertilizers significantly influenced the water content of wheat grains at the 1% and 5% probability levels, respectively. Phosphorus rate had the most (*F* = 11.53) but, the iron rate had the least (*F* = 10.24) influences on the water content. The interaction effect phosphorus and foliar iron fertilizers rate on the water content was not significant (*p* > 0.05) (Table 2).

**Table 2:** Results of analyses of variance (Mean Square Error) for the quality parameters of wheat grains as affected by phosphorus and iron fertilization rate

Source of variation	Dependent variable	*df*	Mean square error	*F* Value
Iron rate (IR)	Protein content	2	12.198	480.009^a^
	Fiber content	2	6.663	34.664^a^
	Water content	2	0.444	10.237^b^
Phosphorus rate (PR)	Protein content	3	5.981	235.362^a^
	Fiber content	3	8.278	43.070^a^
	Water content	3	0.500	11.530^a^
IR ×PR	Protein content	6	0.138	5.430^a^
	Fiber content	6	0.085	0.440^ns^
	Water content	6	0.144	3.331^ns^
Error	Protein content	24	0.025	
	Fiber content	24	0.192	
	Water content	24	0.043	

^a^Significant at 1% level, ^b^Significant at 5% level, ^ns^not significant.

### 3.1 Effect of phosphorus rate

The results of mean comparison for wheat grains quality parameters based on Duncan's multiple test ranges at different levels of levels of phosphorus and iron fertilization are presented in Table 3. It is seen from the data in Table 3 that the protein content of wheat grains increased by increasing the phosphorus fertility rate. The result shows, as the rate of chemical fertilization has significant effects on the physicochemical, mechanical, and thermal properties of seed/grains, it also has an important effect on the seed/grains quality parameters. In addition, the result confirms that the dose of phosphorous fertilization has a significant effect on the chemical compositions of wheat grain. It is generally believed, however, that phosphorus slightly affects grain proteins in winter wheat (Batten, Fettell, Mead, & Khan, 1999; Benbella & Paulsen, 1998; Gaj, Górski, & Przybyl, 2013). Berecz (2001) pointed out that the influence of phosphorus fertilization on wheat quality characters dependent largely on the nitrogen and phosphorus rates (Gaj et al., 2013). The issue of interaction between nitrogen and phosphorus is referred to in many publications (Gaj et al., 2013; Kim et al., 2003; Prystupa, Savin, & Slafer, 2004; Sadras, 2006). Rahim, Ranjha, and Waraich (2010) reported that protein content in wheat grains increased at each increment of phosphorus fertilizer. Also, Rasul (2016) reported that the highest mean values (8.50%) of protein content in wheat seed were recorded in 250 kg/ha in comparison with all other treatments (0, 150, 200 kg/ha). The results reported by Gaydou and Arrivets (1983) and Israel, Kwanyuen, Burton, and Walker (2007) showed that phosphorus fertilization increase oil and protein contents in the soybean seed. The results disagree with the results reported by Popovic (2015). He observed that the protein content of wheat seed increased as phosphorus amounts decreased in the trials.

**Table 3:** The results of mean comparison for wheat grains quality parameters based on Duncan's multiple tests ranges at different levels of levels of phosphorus and iron fertilization

Independent variable	Dependent variable (wheat grain quality parameters)		
	Protein content (%)	Fiber content (%)	Water content (%)
Phosphorus rate (kg/ha)			
0	13.56 c^a^	13.76 a	7.36 b
75	14.29 b	13.83 b	7.78 a
150	15.53 a	11.70 c	7.30 b
225	14.57 a	11.86 c	7.28 b
Foliar iron rate (L/ha)			
0	13.47 c	13.40 a	7.57 a
1.2	14.52 b	12.19 b	7.51 a
2	15.48 a	12.03 b	7.21 a

a Different letters in column section imply statistically significant differences at the significance level *p* = 0.05.

As shown from the data in Table 3, by increasing the dose of phosphorous from 0 to 150 kg/ha the average values of the protein content of wheat grains significantly increased by 1.14 times (from 13.56% to 15.53%) however, by more increasing the dose of phosphorus from 150 to 225 150 kg/ha, caused a significantly decreased trend in the amount of protein. According to the results of numerous studies, there is a certain optimum level of each fertilizer and for each crop, at which maximum quality parameters such as protein content occurs. So, in the current study, the optimum level of phosphorus fertilizer for wheat was obtained at about 150 kg/ha. The mean values for the protein content of wheat grains were found to be 13.56%, 14.29%, 15.53%, and 14.57% for phosphorus fertilizer rates of 0, 75, 150, and 225 kg/ha, respectively. In addition, the mean values of the protein content of wheat grains at different levels of phosphorus were statistically different from each other (*p* < 0.05), according to Duncan's multiple range test results, (Table 3).

For the fiber content of wheat grains (NDF), the effect of phosphorus fertilizer rate was significant at 1% level (Table 2). It is evident from the data in Table 3 that the fiber content of wheat seeds decreased with an increase in phosphorus fertilizer rate. By increasing the rate of phosphorus from 0 to 225 kg/ha the average values of the fiber content of grains decreased significantly from 13.76% to 11.86%. The average values for the fiber content of wheat seeds were found to be 13.76%, 12.83%, 11.70%, and 11.86% for phosphorus fertilizer rates of 0, 75, 150, and 225 kg/ha, respectively. Moreover, according to Duncan's multiple range test results, the mean values of the fiber content of seeds at 0 and 75 kg/ha, rates of phosphorus were statistically different from 150 and 225 kg/ha (*p* < 0.05) (Table 3). The phosphorus fertilizer rate of 0 kg/ha gave the highest mean percentage of the fiber of 13.76%. No reported result obtained in the published literature for the effect of phosphorus fertilizer rate on the fiber content of whole wheat grain to compare with the results obtained in this research.

The effect of phosphorus fertility level on the water content (stored water for 6 months) of wheat grains was significant at the 1% probability level (Table 2). The result of the mean comparison in Table 3 indicated that by increasing the rate of phosphorus from 0 to 75 kg/ha the average values of the water content of wheat grains significantly increased from 7.36% to 7.87% however, further increase in phosphorus 75–225 kg/ha caused a significantly decreased trend in the amount of water from 7.78% to 7.28%. The mean values for the water content of wheat grains were found to be 7.36%, 7.78%, 7.30%, and 7.28% for phosphorus fertilizer rates of 0, 75, 150, and 225 kg/ha, respectively. Moreover, The result of the mean comparison showed that the mean value of the water content of seeds at 75 kg/ha rate of phosphorus was statistically different from other rates (*p* < 0.05 (Table 3).

Regression analysis was used to find fit the best general models to the data for the relationship between the quality parameters in wheat grain and phosphorus fertilizer rate. The result showed that the quality characteristics of wheat seed include protein, fiber, and water contents were polynomial (quadratic) functions of phosphorus fertilizer rate and there was a positive relationship between phosphorus and content of protein but negative for the contents of fiber and water. The relationships support the hypothesis on the effect of phosphorus on grain quality. Therefore, the dependence of protein content (*PC*, %), fiber content (*FC*, %) and water content (*WC*, %) of wheat seed on the rate of phosphorus fertilizer (*P,* kg/ha) was expressed by the following best‐fit equations:

(1) $$PC=0.005P^{2}+0.0238P+13.75\;R^{2}=0.987$$

(2) $$FC=-0.004P^{2}+0.0271P+13.5\;R^{2}=0.999$$

(3) $$WC=-0.0002P^{2}+0.0176P+7.36\;R^{2}=0.998$$

### 3.2 Effect of foliar iron rate

The mean comparison results showed that (Table 3) foliar application of iron increased the wheat grain protein content over control (untreated sample plants). The values of grains protein content had a significantly increasing trend by 1.05 times (from 14.95% to 15.74%) with an increase in the foliar iron fertilizer rate from 0 to 2 L/ha (Table 3). The mean values for the percent protein of wheat grains were found to be 14.95%, 14.89%, and 15.74% for iron fertilizer rates of 0, 1.2, and 2 L/ha, respectively. Moreover, according to Duncan's multiple range test results, the mean values of the protein content of seeds at the 2 L/ha iron fertilizer rate was statistically different (*p* < 0.05) from other rates (0 and 1.2 L/ha) (Table 3). The result is similar to the results reported by Ali (2012). He observed that the protein content of wheat seed increased as the foliar application of iron increased. The result indicating that the iron fertilizer such as phosphorus had a significant effect on the chemical composition of wheat grain. This may be due to the importance of iron for the formation of chlorophyll, enzyme and photosynthesis systems and respiration of plants (Ali, 2012; Havlin et al., 2005).

For the fiber content of grains (NDF), it is evident from the data in Table 3 that the fiber content of from wheat grains decreased with an increase in foliar iron fertilizer rate. This was an interesting result, but unfortunately, the effect of iron fertilization rate on the fiber content in wheat grains has not been defined unambiguously in literature, to compare with the results obtained in this research. By increasing the rate of foliar iron fertilization from 0 to 2 L/ha, the average values of the fiber content of grains decreased significantly from 13.63% to 12.57%. The mean values of the fiber content of wheat grains were found to be 13.63%, 13.16%, and 12.57% for foliar iron fertilizer rates of 0, 1.2, and 2 L/ha, respectively. The mean comparison results showed that the mean values of the fiber content of wheat grains at the different rates of iron fertilization were statistically different from each other (*p* < 0.05), based on Duncan's multiple range tests (Table 3).

For the water content of wheat seeds, the effect of foliar iron fertility level was significant at 5% level (Table 2). From the data in Table 3, it is seen that the water content of wheat grains decreased with an increase in the rate of foliar iron fertilizer. By increasing the rate of foliar iron from 0 to 2 L/ha the mean values of the water content of wheat grains decreased significantly from 7.57% to 7.21%. The average values for the water content of wheat seeds were found to be 7.57%, 7.51%, and 7.21% for foliar iron fertilizer rates of 0, 1.5, and 2 L/ha, respectively. No reported results for the effect of fertilization rate of foliar iron on the water contents of seeds were found to compare with the results obtained in this study.

Regression analysis on the relationship between the quality parameters in wheat grains showed that the content of protein increased as a linear (positive relationship), but fiber and water contents decreased as polynomial functions (negative relationship) of the foliar iron fertilizer rate. The relationships supported the hypothesis on the effect of foliar iron on the wheat grain quality. The following best‐fit equations were obtained for the relationship between protein content (*PC*, %), fiber content (*FC*, %) and water content (*WC*, %) of wheat grains and the rate of foliar iron fertilizer (*Fe,* L/ha):

(4) $$PC=0.975Fe+13.845\;R^{2}=0.9982$$

(5) $$FC=0.3991Fe^{2}-1.4803Fe+13.395\;R^{2}=0.9998$$

(6) $$WC=-0.1625Fe^{2}+0.1454Fe+7.57\;R^{2}=0.9998$$

### 3.3 Interaction effect of phosphorus and iron rate

**Figure 1:**
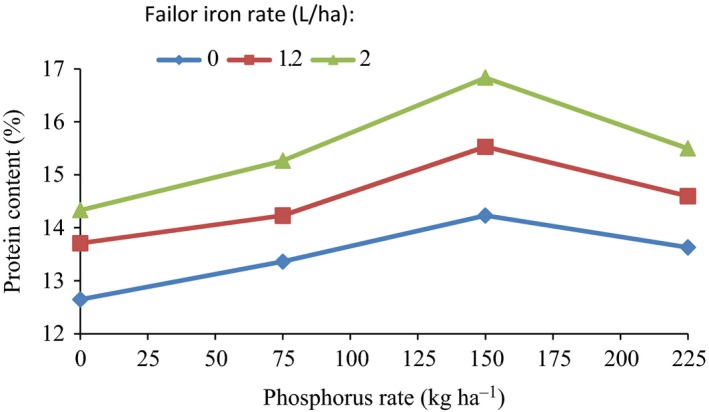
Wheat grain protein content variation with phosphorus fertilizer rate at different foliar iron fertilizer rates

Statistical analysis showed that the effect of the interaction between phosphorus and iron fertilizers rate on the protein content of wheat seeds was significant (*p* < 0.05) (Table 2). The values of the protein content of whole wheat grains in the interaction between fertilization of phosphorus and foliar iron are presented in Figure 1. The data for the protein content of grains in Figure 1 varied from 12.65% to 16.83%. The minimum value (12.65%) was obtained for the 0 kg/ha phosphorus with 0 L/ha iron rate (control treatments). The maximum value (16.83%) obtained for the interaction of 150 kg/ha rate of phosphorus and 2 L/ha iron rate iron fertilization. As follows from the Figure 1, at all foliar iron rates, the protein content of seeds increased with increase in the rate of phosphorus from 0 to 150 kg/ha, however, further increase in phosphorus 150–225 kg/ha, caused a non‐significant decreased trend in the amount of protein content. The rate of change in protein content of grains by an increase in the rate of phosphorus is not the same for all the levels of iron fertilizer. The effect of fertilization level of phosphorus on the protein content of the seeds is stronger at higher iron rats than at lower ones.

At 0 L/ha iron rate, the protein content of seeds increased from 12.65% to 14.23% (by 1.12 times) with an increase in the phosphorus rate from 0 to 150 kg/ha. Corresponding values of protein contents were from 13.70% to 15.53% (by 1.13 times) and from 14.33% to 16.83% (by 1.17 times) for the same phosphorus rate, at 1.2 and 20 L/ha iron rates, respectively.

The wheat seeds protein content was related to the phosphorus fertilizer rate in the range of 0–225 kg/ha, at different foliar iron fertilizer rates, by regression analysis. The results showed that the protein content of seeds increased as polynomial functions with an increase in the rate of phosphorus, at all the iron rates considered. The equations representing the relationship between the percentage protein content (*PC*) of seeds and phosphorus rate (*P*), at different iron rates, and their coefficients of determination (*R* ^2^) are presented in Table 4.

**Table 4:** Equations representing the relationship between protein content (%) of wheat seeds and phosphorus fertilizer rate at different foliar iron rates

Foliar iron rate (L/ha)	Equation	*R* ^2^
0	*PC *= 0.0004 *P* ^2^ − 0.0075 *P *+ 14.333	0.999
1.2	*PC* = 0.0003 *P* ^2 ^− 0.0116 *P* + 13.706	0.999
2	*PC *= 0.0002 *P* ^2^ − 0.0012 *P* + 12.6467	0.999

*PC = *protein content (%), *P = *phosphorus rate (kg/ha).

**Figure 2:**
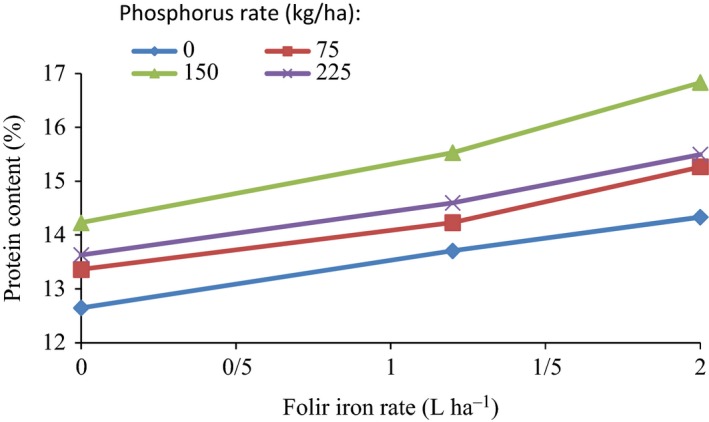
Wheat grain protein content variation with foliar iron fertilizer rate at different phosphorus fertilizer rates

Figure 2 shows the protein content of wheat seeds according to the rate of iron fertilizer at different fertilization rates of phosphorus. As follows from the Figure 2 the percentage protein content of seeds increases with an increase in the rate of foliar iron rates, at all the phosphorus rates. However, the rate of increase in the protein of seeds by an increase in the rate of foliar iron is not the same for all the levels of phosphorus fertilizer. The effect of fertilization rate of iron on the increasing the content of protein of wheat grain is stronger at higher phosphorus rats (150 kg/ha) than at lower ones. At 0 phosphorus rate, the percentage protein of seeds increased from 12.65% to 14.33% (by 1.13 times) with an increase in the iron rate from 0 to 2 L/ha. Corresponding percentage changes in the content of protein were from 13.36% to 15.27% (1.14 times), 14.23% to 16.83% (1.18 times) and from 13.63% to 15.80% (1.13 times) for the same iron rate, at 75, 150, and 225 kg/ha phosphorus rates, respectively. The protein content of whole wheat grains was related to the foliar iron fertilization rate in the range of 0–2 L/ha, at different phosphorus fertilizer rates, by regression analysis. The results showed that the protein content of seeds increased linearly with an increase in the rate of iron fertilizer, at all the phosphorus rates considered. The equations representing the relationship between percentage protein content of wheat seeds (*PC*) and iron fertilizer rate (*Fe*), at different phosphorus rates, and their coefficients of determination (*R* ^2^) are presented in Table 5.

**Table 5:** Equations representing the relationship between protein content (%) of wheat grains and foliar iron fertilizer rate at different phosphorus rate

Phosphorus rate (kg/ha)	Equation	*R* ^2^
0	*PC *= −13.75 *Fe* ^2^ + 12.75 *Fe* + 66.18	1
75	*PC *= −14.76 *Fe* ^2^ + 13.58 *Fe* + 60.18	1
150	*PC* = −5.247 *Fe* ^2^ − 2.739 *Fe *+ 47.40	1
225	*PC *= −13.01 *Fe* ^2^ + 3.31 *Fe *+ 35.80	1

*PC *= protein content (%), *Fe* = iron rate (L/ha).

## 4 CONCLUSIONS

The results of this study indicated that the phosphorus and iron fertilizers had significant effects on the composition of whole wheat grains, increased their protein content, and reduced the fiber and water contents. The key results that can be noted are: The effect of phosphorus fertilizer rate on the increased protein and reducing fiber and water contents of wheat seeds was higher than the effect of iron fertilizer rate. Increasing the phosphorus fertilizer rate from 0 to 150 kg/ha caused a significant increase in the average values of protein content from 13.56% to 15.35%. The wheat grains have higher protein content with phosphorus supply, with the best dose of the application being 150 kg/ha as there was no further increase in protein content with 225 kg/ha. By increasing the rate of the foliar iron rate from 0 to 2 L/ha the average values of the protein content of seeds significantly increased as a linear relationship by 1.5 times (from 13.47% to 15.48%). As the rates of phosphorus and iron fertilizers increased from 0 to 225 kg/ha and from 0 to 2 L/ha, the fiber content of wheat grain decreased significantly from 13.76% to 11.86% and from 13.40% to 12.03%, respectively. These changes were from 7.36% to 7.28% and from 7.57% to 7.21%, respectively, for water content.

## CONFLICT OF INTEREST

The authors declare that there are no conflicts of interest. Furthermore, the manuscript does not contain experiments using animals or human.
